# *GSTP1* and *GSTO1* Variant Alleles Affect Susceptibility to *Helicobacter pylori* Infection and Severity of *Helicobacter pylori*-Associated Clinical Manifestations †

**DOI:** 10.3390/ijms26020488

**Published:** 2025-01-09

**Authors:** Ivana Pantic, Sofija Lugonja, Djurdja Jerotic, Marija Pljesa-Ercegovac, Marija Matic, Nikola Bakovic, Marko Vojnovic, Tatjana Simic, Tamara Milovanovic, Ana Savic-Radojevic

**Affiliations:** 1 Clinic for Gastroenterology and Hepatology, University Clinical Center of Serbia, 11000 Belgrade, Serbia; ilic.ivana04@gmail.com (I.P.); bakovicnikola27@gmail.com (N.B.); marko.vojna@gmail.com (M.V.); 2 Division of Gastroenterology, Department of Internal Medicine, General Hospital “Djordje Joanovic”, 23000 Zrenjanin, Serbia; prolesofija@gmail.com; 3 Faculty of Medicine, University of Belgrade, 11000 Belgrade, Serbia; djurdja.jovanovic@med.bg.ac.rs (D.J.); m.pljesa.ercegovac@gmail.com (M.P.-E.); marija.matic@med.bg.ac.rs (M.M.); tatjana.simic@med.bg.ac.rs (T.S.); 4 Institute of Medical and Clinical Biochemistry, 11000 Belgrade, Serbia; 5 Center for Excellence for Redox Medicine, 11000 Belgrade, Serbia; 6 Department of Medical Sciences, Serbian Academy of Sciences and Arts, 11000 Belgrade, Serbia

**Keywords:** *Helicobacter pylori* (HP), antioxidant gene variants, GSTP1, GSTO1, GSTO2

## Abstract

Considering the mutual relationship between redox disbalance and inflammation in *Helicobacter pylori* (HP) infection, we aimed to evaluate whether the polymorphisms in antioxidant glutathione transferases genes (*GSTP1* rs1695, *GSTP1*rs1138272, *GSTO1* rs4925 and *GSTO2* rs156697) modify susceptibility to HP infection, as well as the severity of HP-associated gastric manifestation development. Therefore, GST gene polymorphisms were determined via the appropriate PCR in 101 HP-positive and 107 HP-negative patients. Our results show that carriers of the GSTP1*G/G variant genotype (rs1695) or at least one GSTP1*T variant allele (rs1138272) were more prone to the development of HP-positive gastritis compared with reference allele carriers (OR = 3.21, 95%CI = 1.15–8.91, *p* = 0.025 and OR = 2.31, 95%CI = 1.14–4.89, *p* = 0.021, respectively), which was confirmed by haplotype analysis. HP-positive carriers of the GSTO1*A variant allele showed increased risk of developing gastric atrophy and precancerous gastric lesions compared with the reference one (OR = 2.49, 95%CI:1.04–5.96, *p* = 0.04 and OR = 2.98, 95%CI = 1.21–7.34, *p* = 0.018, respectively). HP-positive carriers of the GSTO2*G variant allele were less prone to developing moderate/severe inflammatory infiltration (OR = 0.35, 95%CI = 1.04–5.96, *p* = 0.04), whereas the GSTP1*T variant allele was significantly associated with active inflammation (OR = 4.09, 95%CI = 1.04–5.96, *p* = 0.042). In conclusion, antioxidant GST genetic propensity seems to have an important impact on both acute and chronic forms of HP infection.

## 1. Introduction

The prevalence of *Helicobacter pylori* (HP) infection still exceeds more than half of the worldwide population, showing significant variation among regions, as the highest prevalence has been reported in Africa (79.1%), while the lowest infection rates have been reported in North America and Oceania (37.1% and 24.4%, respectively) [1]. In addition to bacterial virulence factors, the multifaceted interplay between the host’s genetic factors and immune response, as well as different environmental and lifestyle factors, contributes to the HP clinical presentation spectrum [2]. In that setting, despite known gastrointestinal clinical diversity, including chronic active gastritis, peptic ulcer disease, gastric extranodal marginal zone lymphoma of mucosa-associated lymphoid tissue (MALT) and gastric adenocarcinoma, special clinical attention is primarily paid to the mechanisms contributing to long-term HP infection [2,3].

HP virulence factors and their interaction with different components of the immunological response have been extensively studied both in vitro and in vivo [4,5,6]. Accordingly, the accumulation of neutrophils and other immune cells in the *lamina propria,* followed by the production of interferon-γ (IFN-γ), tumor necrosis factor α (TNF-α) and interleukin-1 (IL-1β), leads to prolonged inflammation, oxidative distress and subsequent damage to the gastric mucosa [2,7]. The conversion of cytokine precursors into mature IL-1β and IL-18 is mediated by the NLRP3 inflammasome, as one of the most important components of innate immunity [8]. It seems that during gastrointestinal inflammation, the activation of inflammasome might have both beneficial and adverse effects [2,3]. Moreover, some in vitro studies have shown that HP has the ability to prevent the activation of this inflammasome [9]. Therefore, the level of inflammasome activity might represent an important mechanism underlying the long-term bacterial colonization of the gastric mucosa.

Considering the involvement of redox disbalance in the pathogenesis and manifestations of HP infection [7,10,11,12], it seems that polymorphisms in antioxidant enzymes could modify both the onset and the course of infection. The polymorphic family of cytosolic glutathione transferases (GSTs) are important for the maintenance of redox homeostasis and the modulation of numerous intracellular signaling pathways [13]. In context of HP infection, only a few studies evaluated the role of GSTs and oxidative metabolism during recent decades, such as the study by Tatemichi M et al., which investigated the association between several *GST* polymorphisms and IgG levels in serum against HP in 300 HP-seropositive subjects, as well as the study by Shirin H et al., which intended to measure the levels of reduced glutathione in the context of HP infection [14,15]. However, more recent data have underlined the significance of not only antioxidant but also signaling regulatory roles of GSTs, which could be both included in the disease pathophysiology. It has been reported that GSTO1 achieves its proinflammatory action as an NLRP3 inflammasome activator by the deglutathionylation of the NEK7 (NIMA-related kinase) molecule, which subsequently results in the production of IL-1β [16]. Concerning *GSTO* gene polymorphisms, the significance of two single-nucleotide polymorphisms (SNPs) has been studied in various malignant and non-malignant conditions: *GSTO1* rs4925 and *GSTO2* rs156697 [17,18,19,20,21]. It is now known that the GSTO1*C419A (rs4925) SNP is associated with the reduction in the enzymes’ deglutathionylase activity [17,22,23], while GSTO2*A424G (rs156697) affects enzyme expression [24]. In addition to omega-class GSTs, the role of GSTP1 could also be of significance in HP infection, especially considering its non-catalytic role in the regulation of the mitogen-activated protein kinase (MAPK) signaling pathway involved in apoptosis and cellular proliferation [25]. Specifically, the monomeric form of GSTP1 inhibits the activation of the MAPK signaling molecule, JNK (c-Jun N-terminal kinase), by protein–protein interaction [25]. In this specific way, GSTP could have an impact on modulating the activity of transcription factor nuclear factor-κB (NF-κB) [26]. When it comes to genes encoding GSTP, two SNPs have gained increasing attention, *GSTP1* rs1695 and *GSTP1* rs1138272, both of which have been described to lead to altered catalytic and signaling activity of GSTP1 [26].

Given the limited data concerning the peculiarities of the host’s immune response and its influence on the onset and progression of HP infection and the possible functional significance of the most common polymorphisms in genes encoding GSTO and GSTP class members, we aimed to evaluate the effect of the *GSTO* and *GSTP* genotypes on individual susceptibility to HP infection and their association with clinical presentation, along with relevant socio-epidemiological factors.

## 2. Results

### 2.1. Demographic and Clinical Characteristics

The demographic characteristics, environmental risk factors and the clinical characteristics of HP-negative and HP-positive patients are presented in Table 1. There were no statistically significant differences in age, sex, obesity, hypertension (HTA) and type 2 diabetes (T2D) prevalence, smoking status and alcohol use between the investigated groups. In contrast, ASA use was statistically more common in HP-negative compared with HP-positive patients (26% vs. 14%, *p* = 0.020). When it comes to clinical characteristics, HP-positive patients had worse findings for each analyzed endoscopic and histological feature (*p* < 0.05). No statistically significant differences in complete blood count parameters were noted (*p* > 0.05).

### 2.2. Distribution of GSTO1 rs4925, GSTO2 rs156697, GSTP1 rs1695 and GSTP1 rs1138272 Genotypes Among HP-Negative and HP-Positive Patients

The GST genotype distribution within the HP-negative and HP-positive is presented in Table 2. The analyses showed that individuals carrying the homozygous GSTP1*G/G rs1695 variant genotype exhibited three times higher odds (OR = 3.21, 95%CI = 1.15–8.91, *p* = 0.025), while carriers of at least one GSTP1*T rs1138272 variant allele had two times higher odds of developing HP-positive gastritis (OR = 2.31, 95%CI = 1.14–4.89, *p* = 0.021) compared with those carrying reference alleles. According to our analysis, the *GSTO1* and *GSTO2* polymorphisms did not exhibit any influence on the risk of HP-positive gastritis development.

Additional *GSTO* (*GSTO1* rs4925/*GSTO2* rs156697) and *GSTP1* (rs1695/rs1138272) haplotype analyses yielded similar results, which are presented in Table 3. As shown, carriers of the H3 haplotype, comprising GSTP1*G and GSTP1*T variant alleles, presented a more than 3-fold higher risk of HP-positive gastritis development (OR = 3.12, 95%CI = 1.29–7.67, *p* = 0.012), while the *GSTO* haplotype did not exert significant influence.

### 2.3. Association Between GST Polymorphisms and Histological Findings in HP-Negative Patients

**Figure 1:**
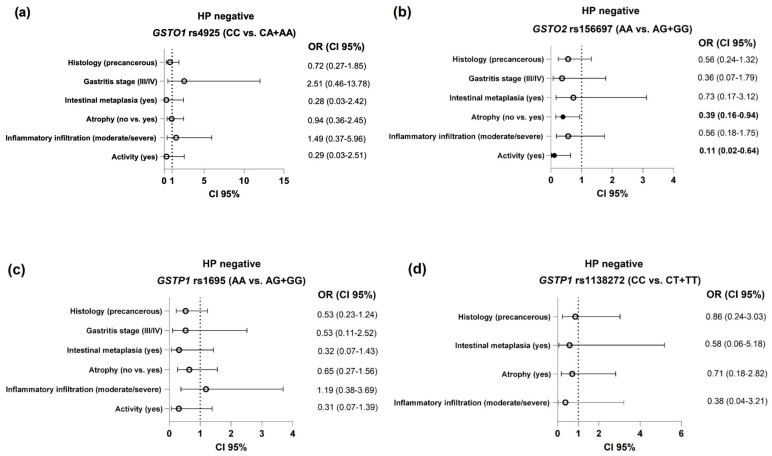
The association between the *GST* polymorphisms ((**a**)—*GSTO1* rs4925; (**b**)—*GSTO2* rs156697; (**c**)—*GSTP1* rs1695; (**d**)—*GSTP1* rs1138272) and the evaluated histological features in HP-negative subjects. Analysis adjusted for age, gender, alcohol use, smoking status, ASA, and NSAID use.

The risk of development of specific histological findings in HP-negative patients with respect to the *GST* genotypes is presented as a tree plot (Figure 1). As presented, no significant association was noted between the *GSTO1* rs4925, *GSTP1* rs1695 or *GSTP1* rs1138272 polymorphisms and the evaluated clinical features in the HP-negative group (Figure 1a,c,d). On the other hand, carriers of at least one GSTO2*G variant allele had decreased odds of developing gastric atrophy (OR = 0.39, 95%CI = 0.16–0.94, *p* = 0.04) and active inflammation (OR = 0.11, 95%CI = 0.02–0.64, *p* = 0.013) than GSTO2*A reference carriers (Figure 1b).

### 2.4. Association Between GST Polymorphisms and Endoscopic and Histological Findings in HP-Positive Patients

**Figure 2:**
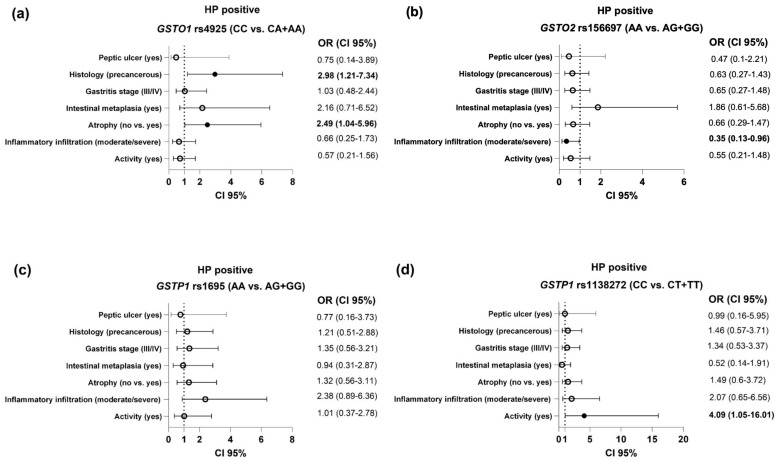
The association of the *GST* polymorphisms ((**a**)—*GSTO1* rs4925; (**b**)—*GSTO2* rs156697; (**c**)—*GSTP1* rs1695; (**d**)—*GSTP1* rs1138272) and the evaluated endoscopic and clinical findings in HP-positive patients. Analysis adjusted for age, gender, alcohol use, smoking status, ASA, and NSAID use.

The association between the *GST* polymorphisms and the evaluated clinical features of the HP-positive group is presented in Figure 2. It is important to note that carriers of at least one GSTO1*A variant allele presented an up to three times higher risk of developing gastric atrophy and precancerous gastric lesions (gastric atrophy and/or intestinal metaplasia), compared with GSTO1*C reference carriers (OR = 2.49, 95%CI = 1.04–5.96, *p* = 0.04 and OR = 2.98, 95%CI = 1.21–7.34, *p* = 0.018, respectively), as shown in Figure 2a. Considering inflammation parameters, carriers of the GSTO2*G variant allele were at decreased risk when it came to the development of moderate/severe inflammatory infiltration (OR = 0.35, 95%CI = 0.13–0.96, *p* = 0.04), whereas carriers of the GSTP1*T variant allele genotype exhibited a 4-fold higher risk of developing active inflammatory infiltrate (OR = 4.09, 95%CI = 1.05–16.01, *p* = 0.042) compared with carriers of the reference allele (Figure 2c).

## 3. Discussion

This case–control study aimed to evaluate the role of genetic polymorphisms of antioxidant enzymes in HP infection susceptibility and its clinical presentation. Based on the mutual relation between the dysregulation of redox homeostasis and inflammation in the course of HP infection, we have proposed a modifying effect of polymorphisms in antioxidant enzymes (*GSTP1* rs1695, *GSTP1* rs1138272, *GSTO1* and *GSTO2*) on individual susceptibility to the development of HP-associated clinical manifestations. The data obtained herein have shown significant impact of the analyzed polymorphisms on the risk of development of HP-associated clinical manifestations. Specifically, carriers of at least one rs1138272 GSTP1*T variant allele exhibited an over four-fold higher risk of developing active inflammatory infiltrate among HP-positive patients, whereas moderate/severe infiltration of inflammatory cells was related to the GSTO2*A reference allele. It is important to note that HP-positive carriers of at least one GSTO1*A variant allele presented an up to three times higher risk of developing gastric atrophy and precancerous gastric lesions (gastric atrophy and/or intestinal metaplasia), compared with the GSTO1*C reference allele.

There are several proposed mechanisms by which polymorphisms of glutathione transferases might affect clinical manifestations caused by HP. As the first line of the enzymatic antioxidant defense system, GSTs significantly contribute to preserving redox homeostasis and the activity of redox-sensitive signaling pathways. Beyond their antioxidant function, GSTs might affect signaling cascades activated by HP, especially NF-κB and MAPK signaling cascades. In gastric epithelial cells, the activation of those signaling pathways is responsible for the production of cytokines and chemokines [27]. Recently, it has been shown that the activity of the ERK1/2 and JNK–MAPK signaling pathways is responsible for increased activity of the metalloproteinases MMP-3 and MMP-9, as important mediators of HP colonization [28]. The results obtained in our study show that the presence of at least one GSTP1*T variant allele (rs1138272) or homozygous GSTP1*G/G variant genotype (rs1695) increases the odds of developing HP colonization and HP-associated clinical manifestations. These findings were confirmed after haplotype analysis, showing that carriers of the H3 haplotype, comprising the GSTP1*G and GSTP1*T variant alleles, are at the highest risk of HP-associated clinical manifestations. The *GSTP1* haplotype with both variant alleles exhibits lower antioxidant potential [29] and, conversely, more efficient inhibition of JNK–MAPK [30,31]. Specifically, GSTP1 acts as a negative regulator of JNK-dependent signaling by the aforementioned GSTP1–JNK interaction. However, even though ROS increase NF- κB expression in diseased tissues, one should bear in mind that the GSTP1–JNK complex tends to degrade in the setting of ROS accumulation [31], which could be considered a hallmark of HP infection, underscoring the dual role of ROS in HP infection. HP-related oxidative stress is mediated by numerous mechanisms [32], one of which could be decreased GST antioxidant function due to the described SNPs. Moreover, ROS could be produced in much lower levels in epithelial cells, compared with phagocytic cells, and therefore play the main role in redox-sensitive signaling, rather than HP eradication [7]. Based on our results, carriers of GSTP1 variant alleles are more prone to HP-associated disease development. What is more, in the HP-positive group, carriers of the GSTP1* T (rs1138272) variant allele exhibited higher odds of developing active inflammatory infiltrate compared with the carriers of the reference one. One of the additional possible explanations might be the recently observed leukotriene synthase activity of GSTP1, which seems to influence chemotaxis and the activation of leukocytes [33]. Given the aforementioned, the effect of GSTP1 polymorphisms on HP susceptibility may be mainly due to its reduced antioxidant function, rather than more efficient JNK–MAPK inhibition, and the consequent reduction in NF-κB activation. Interestingly, the role of GSTP1 has mainly been evaluated in the context of gastric carcinogenesis, with conflicting results [34,35,36], probably as a result of different study designs and examined populations. Still, several studies did report the *GSTP1* variant allele as constituting a risk of gastric carcinogenesis [35,37]. Bearing in mind the Correa cascade, these data could be considered to be in line with our results while underscoring different dominant GSTP1-related molecular mechanisms depending on the infection stage.

According to our analysis, the *GSTO1* rs4925and *GSTO2* rs156697 polymorphisms did not exhibit any influence on the risk of HP-positive gastritis development. On the other hand, we found that in HP-positive patients, carriers of at least one GSTO1*A variant allele had significantly increased odds of developing gastric atrophy and precancerous gastric lesions (gastric atrophy and/or intestinal metaplasia) compared with carriers of the GSTO1*C reference allele. Since the GSTO1*A variant allele exhibits lower deglutathionylation activity in contrast to the reference one [23,38], decreased activation of the NEK7/NLRP3 inflammasome in the course of HP infection might be proposed in those patients. Specifically, the role of the GSTO1 enzyme in the innate immune response is primarily related to the activation of the NLRP3 inflammasome [16]. Besides inflammation and host defense, proposed protective roles of inflammasome activation also include the regulation of cell proliferation and apoptosis, tissue repair, the synthesis of antimicrobial peptides [39], the prevention of tumor development [40] and the regulation of NF-κB and MAPK signaling [41]. In the course of chronic HP infection, recent findings pointed out a protective role of epithelial inflammasomes and IL-18 against the development of preneoplastic changes, whereas IL-1β exhibits a procarcinogenic role, emphasizing their dual functions in gastric pathophysiology [42,43]. It has been proposed that the innate immune response might have a significant role in every step of HP-induced progression, from chronic active non-atrophic gastritis to gastric cancer [44]. Thus, our results on the association between the GSTO1*A variant allele and increased susceptibility to precancerous gastric lesions in HP-positive patients seem to be clinically important, especially for the early recognition of high-risk carriers.

Likewise, the *GSTO2* polymorphism affected susceptibility to HP-associated gastric clinical manifestations. Specifically, we found a significant association between the GSTO2*A reference allele and moderate/severe infiltration of inflammatory cells in HP-positive patients. Similarly, HP-negative patients carriers of the GSTO2*A reference allele had increased odds of developing atrophy and activity of inflammation. It seems that the *GSTO2* polymorphism influences the level of gastric inflammation irrespective of cause. Although we did not find any modifying effect of the *GSTO2* polymorphism on the risk for development of HP-associated gastric pathology, the findings of the present study emphasize the potential incremental value of *GSTO2* genotyping for detecting subclinical dysfunction due to inflammation in those patients. The SNP of the *GSTO2* gene could also affect primarily its antioxidant dehydroascorbate reductase (DHAR) activity [22]. Furthermore, the activity of prolyl hydroxylases, enzymes involved in marking hypoxia-inducible factor-1 (HIF-1α) for ubiquitination and proteasomal degradation, depend on vitamin C as a cofactor [45]. Hypoxia-inducible factor-1 (HIF-1α), representing a key transcriptional regulator of immunity and carcinogenesis, is upregulated by HP in the gastric mucosa even under normoxic conditions. Novel data, however, have identified an important paradox of prolyl hydroxylase inhibitors, which also exert a protective effects against HP-induced gastric inflammation and injury by attenuating the proinflammatory and macrophage immune responses. Specifically, it has been shown that those inhibitors decrease the production of chemokines, the infiltration of neutrophils and the secretion of proinflammatory cytokines, including IL-1β, IL-6 and TNF-α, in HP infection [46]. In this context, it may be hypothesized that the ascorbic acid-dependent inhibition of HIF signaling provides an additional approach to controlling inflammation. These observations align with our findings of the association between *GSTO2* gene variants and different types of gastritis, highlighting the complex interplay of inflammation and redox disbalance in the pathogenesis of this condition.

Finally, the limitations of this study, which refer mainly to the study sample size, should also be addressed. The study sample was based on the number of patients which we were able to recruit, given the specific time during which the study was conducted (Coronavirus-19 disease pandemic). This resulted in limited access to patients, especially HP-positive ones, due to the high antibiotic prescription rates.

Taken together, our results refer to the association between *GSTO1*, *GSTO2* and *GSTP1* genetic variants and HP infection susceptibility, as well as its clinical manifestations. Our results suggest that carriers of at least one *GSTP1* variant allele (rs1695 and rs1138272) present an up to three times higher risk of developing HP infection. Moreover, patients with HP-positive *GSTO1* variant alleles showed increased risk of developing precancerous gastric lesions, while *GSTP1* rs1138272 variant alleles were associated with active inflammation. However, due to the limited sample size, the role of GSTs in HP infection pathophysiology warrants careful interpretation.

### Clinical Relevance and Future Directions

Given that our results suggest that different GST roles might be dominant in different phases of HP infection, further elucidation of the exact roles of GSTs in different HP infection stages (HP colonization, chronic gastritis and carcinogenesis) is needed. Although the role of HP is well described in human gastric diseases, along with the complex underlying molecular mechanisms, further development of different research models is necessary to identify specific therapeutic targets and increase treatment success in all stages of the disease, especially given the increasing microbial resistance and rising prevalence of treatment-resistant HP cases.

## 4. Materials and Methods

### 4.1. Study Population

This prospective case–control study included a total of 218 participants diagnosed and treated at the Clinic of Gastroenterology and Hepatology, University Clinical Center of Serbia, and General Hospital “Djordje Joanovic” in Zrenjanin, between 2020 and 2023. This study included all adult patients in whom esophagogastroduodenoscopy (EGDS) was indicated. The indications for EGDS were as follows: new-onset dyspepsia in patients older than 50 years, presence of “alarm” signs and symptoms (defined as manifest or occult gastrointestinal bleeding, microcytic anemia or sideropenia, unintentional weight loss, dysphagia, palpable stomach mass or abdominal lymphadenopathy), recurrent vomiting, and positive family history of upper gastrointestinal tract malignancies. During the examination, standard gastric biopsies were performed according to the modified Sydney protocol (2 biopsies of the antral and corporal gastric region, placed in separate bottles) [47]. After the pathohistological examination of gastric specimens, all patients with confirmed infection were classified as “HP-positive” and those without detected pathogens in the examined material as “HP-negative”. The exclusion criteria were age (<18 years), limited cognitive abilities, pregnancy or breastfeeding, current or previous malignant disease or immunodeficiency, recent use of antibiotics, presence of inflammatory bowel disease and previous HP or peptic ulcer disease treatment. All patients signed an informed consent form for participation in this study. This study was carried out in accordance with the Helsinki Declaration and was approved by the Ethical Boards of the University Clinical Center of Serbia, General Hospital “Djordje Joanovic” and Faculty of Medicine, University of Belgrade (decision numbers: 760/3, 01-783/04 and 17/I-24, respectively).

### 4.2. Patient Selection

**Figure 3:**
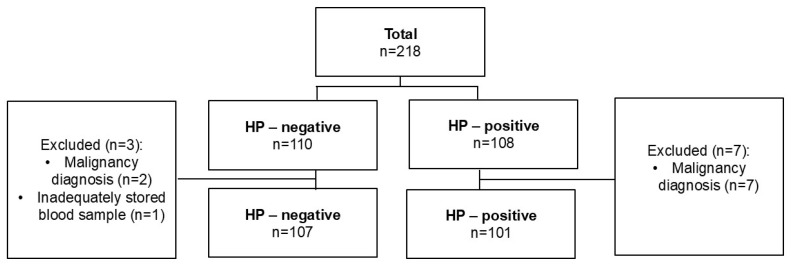
Patients’ selection flowchart.

During the study period, a total of 10 participants were excluded from the research study due to one of the aforementioned exclusion criteria. A total of 208 patients were included in the final analyses (101 cases and 107 controls). The patient inclusion algorithm is presented in detail in Figure 3.

### 4.3. Clinical Characteristics and Questionnaire

Patients’ clinical characteristics were collected from their medical charts. During their gastroenterologist’s appointment, patients were handed a specifically designed questionnaire, which consisted of questions which addressed their socio-demographic characteristics, chief gastrointestinal complaints and the presence of co-morbidities, as well as the use of alcohol, tobacco, non-steroid anti-inflammatory drugs (NSAIDs) and acetyl-salicylic acid (ASA). Participants were not asked to specify which NSAID was used. Complete blood count was obtained from routine laboratory practice.

### 4.4. Endoscopy and Pathophysiology

All patients underwent upper endoscopy using an Olympus endoscopy system (CV-170 processor; Olympus Medical Systems Corporation—Tokyo, Japan). The examination was performed by two experienced endoscopists (S.L. and T.M.). Based on the endoscopic findings, patients were classified into one of the following categories: normal findings, gastritis/gastroduodenitis, erosive gastritis and peptic ulcer disease.

All the collected biopsy specimens were preserved in 10% neutral formalin. Afterward, paraffin-embedded blocks were created. For histological analysis, at least two sets of tissue sections were prepared: one stained with hematoxylin–eosin and the other with Giemsa stain. Pathohistological assessment included the detection of HP and the grading of HP density in the examined material, determining the presence and degree of inflammatory infiltration in the lamina propria and inflammation activity (neutrophil infiltration), as well as the presence of glandular atrophy, intestinal metaplasia and cellular dysplasia. Inflammatory infiltrate density was graded according to the Houston–Sydney system, while the degree of glandular atrophy was staged according to the Operative Link on Gastritis Assessment recommendations (OLGA Staging System) [47,48].

### 4.5. Glutathione Transferase Genotyping

Blood samples were collected in EDTA-coated test tubes for DNA isolation (PureLink™ Genomic DNA Mini Kit; ThermoFisher Scientific, Waltham, MA, USA). The *GSTO1* (rs4925), *GSTO2* (rs156697) and *GSTP1* (rs1695 and rs1138272) polymorphisms were determined by real-time PCR on a Mastercycler^®^ ep realplex platform (Eppendorf, Hamburg, Germany). For this purpose, TaqMan Drug Metabolism Genotyping assays (Life Technologies, Applied Biosystems, Waltham, MA, USA) were used. The assays’ IDs were as follows: *GSTO1* C__11309430_30, *GSTO2* C___3223136_1, *GSTP1* C_3237198_20 and *GSTP1* C_1049615_20.

### 4.6. Statistical Analysis

The analyses of the demographic and clinical characteristics of the study subjects and the association of the assessed gene polymorphisms with the risk of susceptibility of HP infection development, as well as the effect of specific genotypes on its clinical presentation, were performed by using Statistical Package for the Social Sciences (SPSS), ver. 17.0 (Chicago, IL, USA). Differences between the groups were compared by using the χ^2^ test for categorical variables. The χ^2^ test was also used in order to test the deviation of the genotype distribution from the Hardy–Weinberg equilibrium. All categorical variables are presented by using frequency (*n*, %) counts. After the initial test for normality distribution (the Kolmogorov–Smirnov test), Student’s t-test was used to compare continuous variables. All continuous variables are expressed as means ± standard deviations (SDs).

Binary and multinomial logistic regressions were used to assess the contribution of the GST gene polymorphisms to HP-positive gastritis susceptibility, as well as to determine the association between GST polymorphisms and histological findings in both HP-negative and HP-positive patients. Odds ratios (ORs) with 95% confidence intervals (CIs) were computed after adjusting for age, gender, alcohol use, smoking status, ASA use, and NSAID use as the possible confounders.

## Figures and Tables

**Table 1:** Demographic characteristics, environmental risk factors and clinical characteristics of HP-negative and HP-positive patients.

	HP-Negative, *n* = 107	HP-Positive, *n* = 101	*p*
Age (years ± SD)	55.08 ± 15.44	56.1 ± 14.48	0.626
Sex, *n* (%)			
Female	64 (60)	64 (63)	
Male	43 (40)	37 (36)	0.599
Obesity, *n* (%)			
No	87 (81)	81 (80)	
Yes	20 (19)	20 (20)	0.668
HTA, *n* (%)			
No	56 (52)	59 (58)	
Yes	51 (48)	42 (42)	0.378
T2D, *n* (%)			
No	90 (84)	81 (80)	
Yes	17 (16)	20 (20)	0.461
Smoking, *n* (%)			
No	76 (71)	63 (62)	
Yes	31 (29)	38 (38)	0.185
ASA, *n* (%)			
No	79 (74)	87 (86)	
Yes	28 (26)	14 (14)	0.020
NSAID, *n* (%)			
No	61 (57)	63 (62)	
Yes	46 (43)	38 (38)	0.259
Alcohol, *n* (%)			
No	80 (75)	77 (76)	
Yes	27 (25)	24 (24)	0.805
Endoscopic findings, *n* (%)			
Gastritis/gastroduodenitis	98 (92)	85 (84)	
Erosive gastritis	9 (8)	8 (8)	
Peptic ulcer	0 (0)	8 (8)	0.012
Inflammatory infiltrate, *n* (%)			
Mild	91 (85)	27 (27)	
Moderate/severe	16 (15)	74 (73)	<0.001
Inflammation activity, *n* (%)			
No	98 (92)	27 (27)	
Yes	9 (8)	74 (73)	<0.001
Gastric atrophy, *n* (%)			
No	78 (73)	53 (52)	
Yes	29 (27)	48 (48)	0.002
Intestinal metaplasia, *n* (%)			
No	98 (92)	81 (80)	
Yes	9 (8)	20 (20)	0.018
Gastritis stage, *n* (%)			
Stage I/II	99 (93)	53 (53)	
Stage III/IV	8 (7)	48 (47)	<0.001
OLGA stage, *n* (%)			
No atrophy	78 (73)	54 (53)	
Low risk (stage I/II)	29 (27)	40 (40)	
High risk (stage III)	0 (0)	7 (7)	0.002
Histology, *n* (%)			
Normal findings/mild inflammation	61 (57)	11 (11)	
Inflammation	13 (12)	38 (38)	
Atrophy and intestinal metaplasia	33 (31)	52 (51)	<0.001
Peptic ulcer, *n* (%)			
No	107 (100)	93 (92)	
Yes	0 (0)	8 (8)	0.003
NMR ± SD	10.10 ± 5.08	10.22 ± 7.87	0.897
Leu (×10^9^/L) ± SD	7.81 ± 2.79	7.94 ± 2.2	0.723
Neu (×10^9^/L) ± SD	4.95 ± 2.38	4.83 ± 1.99	0.702
Mon (×10^9^/L) ± SD	0.53 ± 0.2	0.58 ± 0.33	0.187
Lym (×10^9^/L) ± SD	2.07 ± 0.82	2.28 ± 1	0.104

HP—*Helicobacter pylori*; SD—standard deviation; HTA—arterial hypertension; T2D—type 2 diabetes; ASA—acetylsalicylic acid; NSAID—non-steroid anti-inflammatory drugs; OLGA—Operative Link for Gastritis Assessment; NMR—neutrophil-to-monocyte ratio; Leu—leukocytes; Neu—neutrophils; Mon—monocytes; Lym—lymphocytes.

**Table 2:** *GSTO1* rs4925, *GSTO2* rs156697, *GSTP1* rs1695 and *GSTP1*rs1138272 genotype distribution in HP-negative and HP-positive patients.

Genotype	HP-Negative, *n* (%)	HP-Positive, *n* (%)	OR (95%CI)	*p*
*GSTO1* rs4925				
*C/C	73 (70)	64 (65)	1 ^a^	
*C/A	21 (20)	21 (21)	1.04 (0.51–2.13)	0.898
*A/A	11 (10)	14 (14)	1.71 (0.69–4.18)	0.241
*C/A + *A/A	32 (30)	35 (35)	1.25 (0.69–2.28)	0.452
*GSTO2* rs156697				
*A/A	38 (36)	43 (43)	1 ^a^	
*A/G	56 (52)	41 (41)	0.65 (0.35–1.21)	0.178
*G/G	13 (12)	17 (17)	1.22 (0.52–2.88)	0.649
*A/G + *G/G	69 (64)	58 (57)	0.76 (0.43–1.36)	0.359
*GSTP1* rs1695				
*A/A	47 (44)	35 (35)	1 ^a^	
*A/G	53 (50)	50 (49)	1.23 (0.68–2.26)	0.486
*G/G	7 (6)	16 (16)	3.21 (1.15–8.91)	0.025
*A/G + *G/G	60 (56)	66 (65)	1.45 (0.82–2.59)	0.203
*GSTP1* rs1138272				
*C/C	92 (87)	74 (73)	1 ^a^	
*C/T	14 (13)	22 (22)	1.86 (0.87–3.95)	0.107
*T/T	0 (0)	5 (5)	/	/
*C/T + *T/T	14 (13)	27 (27)	2.36 (1.14–4.89)	0.021

^a^ Reference group; HP—*Helicobacter pylori*; OR—odds ratio; CI—confidence interval.

**Table 3:** The effect of the GSTO (*GSTO1*rs4925/*GSTO2* rs156697) haplotype and the *GSTP1* (rs1695/rs1138272) haplotype on the risk of development of HP infection.

Haplotype	Polymorphism		HP-Negative %	HP-Positive %	OR (95%CI)	*p*
	*GSTO1* rs4925	*GSTO2* rs156697				
H1	*C	*A	55	55	1.00 ^a^	
H2	*C	*G	25	21	0.82 (0.49–1.39)	0.46
H3	*A	*G	13	16	1.4 (0.69–1.90)	0.61
H4	*A	*A	7	8	1.10(0.51–2.37)	0.81
	*GSTP1* rs1695	*GSTP1*rs1138272				
H1	*A	*C	68	56	1.00 ^a^	
H2	*G	*C	25	28	1.37 (0.79–2.38)	0.26
H3	*G	*T	6	13	3.15 (1.29–7.67)	0.012
H4	*A	*T	1	3	2.24 (0.27–18.30)	0.45

^a^ Reference group; HP—*Helicobacter pylori*; OR—odds ratio; CI—confidence interval.

## Data Availability

The data supporting reported results can be found upon request in the form of datasets available at the Institute of Medical and Clinical Biochemistry, Faculty of Medicine, University of Belgrade.
